# VSELs may obviate cryobanking of gonadal tissue in cancer patients for fertility preservation

**DOI:** 10.1186/s13048-015-0199-2

**Published:** 2015-11-17

**Authors:** Deepa Bhartiya, Sandhya Anand, Seema Parte

**Affiliations:** Stem Cell Biology Department, National Institute for Research in Reproductive Health, JM Street, Parel, Mumbai 400 012 India

**Keywords:** VSELs, Ovary, Testis, Cryopreservation, BMT, Fertility, Cancer

## Abstract

**Background:**

Infertility is an undesirable side effect and gonadal tissue banking is advocated in young cancer patients who are unable to preserve embryos or gametes prior to oncotherapy to achieve biological parenthood later on. Banking gonadal tissue is challenging and protocols to mature gametes *in vitro* are not yet clinically established. Transplanting ovarian cortical tissue at hetero-or orthotopic sites in women and bone marrow transplantation (BMT) in both men and women has resulted in spontaneous recovery of fertility, pregnancy and live births. Various studies in humans and mice suggest that genetic origin of offspring after BMT is similar to transplanted patient and not the donor. Thus the source of oocytes/sperm which result in spontaneous pregnancies still remains contentious.

**Findings:**

Very small embryonic-like stem cells (VSELs) have been reported in adult human testis and ovary, in azoospermic testicular biopsies from survivors of childhood cancer and also in women with premature ovarian failure and menopause. VSELs survive chemotherapy because of their quiescent nature and can be detected in chemoablated mice gonads at protein and mRNA level and also by flow cytometry. Surviving VSELs spontaneously differentiate into oocyte-like structures and sperm when inhibitory factors are overcome *in vitro*. Transplantation of mesenchymal cells (isolated from different sources) has led to regeneration of chemoablated mouse gonads and also live births. Spermatogenesis is also restored from endogenous stem cells on inter-tubular transplantation of Sertoli cells in chemoablated mouse testis.

**Conclusions:**

Endogenous VSELs (which survive oncotherapy) can possibly regenerate non-functional gonads in cancer survivors when exposed to a healthy niche *in vitro* or *in vivo* (by way of transplanting mesenchymal cells which secrete trophic factors required for endogenous VSELs to differentiate into gametes). Presence of VSELs can also explain spontaneous pregnancies after BMT and cortical tissue transplantation (at heterotopic or orthotopic sites). This understanding once verified and accepted by the scientific community could obviate the need to remove whole ovary or testicular biopsy for cryopreservation prior to oncotherapy.

## Background

With advanced treatment options available today, more than 80 % of young cancer patients survive however infertility is often an unwanted side effect. The only option available to them is to cryopreserve the sperm, oocytes or embryos and use later for assisted reproduction to achieve biological parenthood. They could also be counseled to adopt a child. Another option in young children where sperm/oocytes/embryos may not be available is to cryopreserve testicular biopsy or ovarian cortical tissue. One whole ovary is removed surgically and cortical tissue slices are cryopreserved. Later on, when the cancer survivor plans a family, the cryopreserved tissue can be used as a source of gametes for assisted reproduction. Various other strategies which could avoid harming the gonads during oncotherapy like shielding the gonads during radiotherapy and GnRH antagonist are also used as a part of ‘onco-medicine’. The current understanding, clinical practice and American Society of Clinical Oncology (ASCO) guidelines in the field for fertility preservation in cancer patients are available for ready reference [1, 2].

Procedures to obtain gametes from ovarian/testicular tissue or stem cells are not yet clinically established. Spontaneous parenthood followed by live births has been noticed after bone marrow transplantation (BMT) and also on transplanting cortical tissue at hetero- or orthotopic sites. The exact source of sperm or oocyte which results in these spontaneous pregnancies is still not clear. A genetic similarity exists between the patient (recipient) and offspring after BMT refuting earlier claims that transplanted bone marrow could be a source of gametes. Similarly transplanting cortical tissue both subcutaneously or on the surface or vicinity of the non-functional ovary has resulted in live births. What was the source of these oocytes? It could be the transplanted cortical tissue slices or the non-functional gonad regenerated somehow and produced oocytes! Research in authors’ laboratory over last 6–7 years suggests that non-functional gonads possibly regenerate after BMT and cortical tissue transplantation. This is the main focus of the current review which will hopefully result in a major paradigm shift in the field of oncofertility in times to come.

### Stem cells (VSELs) and progenitors (OSCs, SSCs) in ovary and testis

More than a decade ago, Professor Jonathan Tilly from Harvard University, USA challenged the existing dogma that a female is born with a fixed number of eggs at birth; rather he suggested that a continuous replenishment of follicles occurs throughout life [3]. Several groups were drawn to this field of research and evidence was generated that stem cells are housed in adult mammalian ovary surface epithelium (human, sheep, marmoset, rabbit, and mouse) as well as in women with premature ovarian failure (POF) and menopause [4–8]. Presence of stem cells in adult ovary is still not well accepted [9]; however Woods and Tilly [10] have published protocols to isolate ovarian stem cells (OSCs) from human ovaries and equated them to spermatogonial stem cells in testis [11]. Zhang et al. [12] recently described their inability to sort human OSCs using DDX4 as a cell surface marker by compiling together independent work done in four different laboratories to which Woods and Tilly have responded [13]. Silvestris et al. [14] successfully isolated DDX4 positive OSCs and showed further that the DDX4 positive OSCs are also positive for OCT-4. We routinely isolate VSELs and OSCs by mechanical scraping of ovary surface epithelium or by enzymatic method and use flow cytometry only as a tool to further characterize the stem cells [15].

Interesting data has been generated showing that the stem cells in adult ovary can spontaneously differentiate into oocyte-like structures *in vitro* [7, 16, 17] and on injection in human cortical biopsies lead to primordial follicle assembly [18]. A careful examination of the OSE cells smears showed the presence of small (2–5*μ*m), round cells which expressed pluripotent markers SSEA-4, Oct-4, Nanog, Sox-2, and c-kit [5, 6, 19]. Using a polyclonal OCT-4 antibody, Parte et al. [7] for the first time documented the presence of two distinct populations of stem cells in OSE smears including very small stem cells (earlier described by Viran-Klun’s group as well) expressing nuclear OCT-4 and cell surface SSEA-4 termed very small embryonic-like stem cells (VSELs) and slightly bigger stem cells termed ovarian germ stem cells (OGSCs) with cytoplasmic OCT-4. Based on size difference between VSELs (2–5 *μ*m) and OGSCs (>8–10 *μ*m), we speculate that Tilly’s group detected the OGSCs (OSCs) and missed out on the VSELs in adult ovary because of very small size and low abundance of VSELs. Protocols to isolate and characterize both (VSELs and OGSCs) the stem cell populations in adult mammalian ovaries were recently published [15, 20]. Follicle stimulating hormone (FSH) exerts direct action on the ovarian VSELs (express FSH receptors) to undergo self-renewal, clonal expansion to form germ cell nests and further oocyte differentiation [21]. We also discussed that menopause occurs despite high circulatory FSH due to a compromised niche that is unable to support stem cells differentiation into follicles [22]. Stem cells from menopausal ovary spontaneously differentiate into oocyte-like structures when the inhibitory factors are overcome *in vitro* [5, 7]. Similarly stem cells from aged mouse ovaries differentiate and give rise to oocytes on being transplanted into a young somatic environment [23]. Similar VSELs were earlier reported by our group in adult human testis as a sub-group among spermatogonial stem cells (SSCs) on the basis of size and nuclear versus cytoplasmic staining of OCT-4. This was established through extensive characterization by immunolocalization using 3 different OCT-4 antibodies, qRT-PCR studies, in- situ hybridization and Western analysis [24]. VSELs have also been extensively characterized in adult mouse testis [25].

To conclude this section, both ovary and testis harbor pluripotent VSELs along with the specific progenitors which include OSCs in the ovary and SSCs in the testis.

### VSELs are the quiescent stem cell population in the gonads and survive oncotherapy

The VSELs were first reported by Ratajczak’s group [26] in various adult mouse organs including testis and they postulate that pluripotent primordial germ cells (PGCs) during their migration along the dorsal mesentery towards the gonadal ridge to form the germ cells, migrate and settle in various adult organs throughout life [27]. The work became controversial recently when a leading stem cell biologist was unable to detect VSELs in mouse bone marrow [28], but the underlying reasons for their failure were technical as discussed by Ratajczak’s group [29, 30]. We have recently confirmed and extensively characterized VSELs in human cord blood [31]. It is probably because of the very small size, low abundance and minimal cytoplasm that VSELs have remained obscure till now. When cord blood/bone marrow is subjected to Ficoll-Hypaque centrifugation – the VSELs settle down with red blood cells and have been invariably discarded unknowingly in the past [32]. In the gonads, it is relatively easier to conceptualize that a small number of PGCs survive in adult gonads as VSELs and this has also been suggested by other group [33]. Ratajczak’s group have shown that VSELs are relatively quiescent and when mice are subjected to total body irradiation, bone marrow gets depleted of hematopoietic stem cells whereas the VSELs survive and show increased uptake of BrdU [34]. Shin et al. [35] reported that quiescent state of VSELs is because of unique DNA methylation pattern of developmentally crucial imprinted-genes showing hypomethylation/erasure of imprints in paternally methylated genes and hypermethylation of imprints in maternally methylated ones. As a result VSELs express increased levels of H19and Cdkn1c and lowered levels of Igf2 and Rasgrf1accounting for their quiescence.

Available literature suggests that all renewing body organs including skin, hair follicle, gut epithelium, hematopoietic system harbor two populations of stem cells which include quiescent and actively dividing stem cells [36, 37]. Based on these published literature, we decided to study VSELs in mouse ovary and testis to gauge the effect of busulphan and cyclophosphamide treatment on them [8, 25]. Besides immuno-localization and qRT-PCR analysis to show presence of pluripotent VSELs, we were able to quantitate them by flow cytometry as <6 μm sized cells which are LIN-/CD45-/SCA-1+. Results shown in Table 1 demonstrate that VSELs exist in normal gonads, survive chemotherapy and undergo self-renewal in response to PMSG treatment.

**Table 1:** Flow cytometry results on VSELs (LIN-/CD45-/SCA-1+) expressed as % of total cells

	Normal	Chemoablated	PMSG (5 IU) treatment to chemoablated mice
Adult mouse ovary^8^	0.02 ± 0.008 %	0.03 ± 0.017 %	0.08 ± 0.03 %
Adult mouse testis^25^	0.03 ± 0.002 %	0.05 ± 0.005 %	0.1 ± 0.03 %

OCT-4 positive stem cells were also detected by flow cytometry in OSE cells from sheep ovaries (VSELs 1.26 ± 0.19 % of 2–4*μ*m and OGSCs 6.86 ± 0.5 % of 4–9*μ*m). Parte et al. [38] showed that a large number of stem cells are shed on cell culture inserts during both marmoset and human cortical tissue culture and these stem cells undergo spontaneous differentiation into oocyte-like structures *in vitro*. Presence of germ cell nests, Balbiani body-like structures and cytoplasmic streaming extensively described during fetal ovary development, are indeed well recapitulated during *in vitro* oogenesis in adult OSE cultures along with characteristic expression of stem/germ cell/oocyte markers [13]. Similar VSELs have also been reported in testicular biopsy collected from azoospermic cancer survivors [39] and also in POF ovaries [5, 6].

### Testicular function restoration in cancer survivors

Spontaneous paternity and live birth following bone marrow conditioning and transplantation has surprised everyone. Ignatove et al. [40] reviewed 8 such cases and reported two more themselves, where although the patients were azoospermic, testicular sperm helped attain biological parenthood. Dupont et al. [41] recently reported spontaneous conception after autologus BMT. A 20 years old man suffering from acute promyelocytic leukemia, even though was repeatedly diagnosed azoospermic with zero sperm count fathered healthy children spontaneously [41]. The underlying reason is not yet clear but it is likely that there may be a stem cell link for fertility restoration in men similar to that discussed by Oktay et al. [42] in women (please refer to next section).

Transplanting germ cells from cryopreserved testicular tissue still remains experimental and has not been extensively studied in men as yet. Hermann et al. [43] have reported initial success and carried out SSCs transplantation in 18 adult and 5 pre-pubertal busulphan treated Rhesus monkeys through ultrasound guided transfer via rete testis. Sperm collected from these monkeys were injected in 85 oocytes by ICSI, 81 fertilized and 4 cells to blastocyst stage embryos were obtained. 7/81 embryos were of donor origin. Struijk et al. [44] critically reviewed the progress made to restore fertility in survivors of childhood cancer by auto- transplanting SSCs and concluded that transplanted cells colonize but do not differentiate. Navaeian-Kalat et al. [45] reviewed available literature and suggested that trans-differentiation of mesenchymal cells is the only way to treat infertility. Several attempts have been reported to restore spermatogenesis in chemoablated testis by transplanting mesenchymal cells as shown in Table 2. Different doses of mesenchymal cells (and isolated from different sources) have been injected directly into the seminiferous tubules via rete testis or efferent ducts, intravenous injections or direct inter-tubular injections into the testis. It has been postulated that mesenchymal cells are ‘pluripotent’ and can trans-differentiate into gametes. But this is possibly not true [57] and our results suggest that mesenchymal cells only provide a niche (source of growth factors) to the VSELs that survive chemotherapy. Rather than injections into the rete testis or efferent ducts of the seminiferous tubules (challenging technically and not a practical solution), inter-tubular injections of Sertoli cells or mesenchymal cells as a simple OPD procedure may suffice to restore spermatogenesis – based on our studies in mice [25].

**Table 2:** Mesenchymal Cells to Restore Spermatogenesis

Study	Highlights
Hsiao et al., 2015 [46]	Six to eight-week-old Sprague–Dawley rats underwent torsion for 3 h, followed by detorsion on the left testis. 3 × 10^4^ MSCs from human orbital fat tissues (OFSCs) were injected via local injection into the left testis 30 min before detorsion.
	Animals were sacrificed 7 days after torsion-detorsion. Local injections of OFSCs prevented torsion-induced infertility. Serum testosterone secretion was increased, while the elevation of FSH triggered by testicular injury was balanced. OFSCs also produced SCF in the damaged testis. Immunofluorescence staining revealed that most transplanted cells surrounded the Leydig cells. Some of transplanted cells differentiated into p450 expressing cells within 7 days.
Mehrabani et al., 2015 [47]	Rat adipose tissue derived mesenchymal stem cells (ATMSCs) were injected in chemoablated testis (two doses of 10 mg/kg of busulfan with 21 days interval) into the efferent duct of right testes. Seminiferous tubules treated with ATMSCs had normal spermatogenesis whereas the untreated seminiferous tubules were empty.
Abd El Dayem et al., 2015 [48]	Studies were done to restore fertility and reproductive functions after testicular failure induced by butributyltin oxide. Rats were injected a single dose of intravenous MSCs (3×10^6^ cells) and followed up after 5 months. Transplantation of MSCs restored body weight, fertility rate, serum testosterone, LH, FSH hormones, testicular enzymes, sperm counts and improved testicular DNA fragmentation. MSCs transplantation showed normal spermatogenesis and complete recovery in germinal layers. A new therapeutic concept for male infertility treatment.
Zahkook et al., 2014 [49]	MSCs were transplanted (1×10^8^ cells via efferent ducts into the seminiferous tubules) in chemoablated rat induced by busulphan treatment. Some restoration of spermatogenesis was noted associated with increase in size of testis. MSCs have potential to trans-differentiate into germ cells and sperm *in vivo* in testicular microenvironment.
Anand et al., 2014 [25]	Intertubular injection of Sertoli/ bone marrow derived MSCs (1×10^4^ cells) in chemoablated (25 mg/Kg busulphan) mouse testis. Effects were studied after 2 months. The transplanted Sertoli/MSCs aligned as neo-tubules and were a source of growth factors to the VSELs that survived in chemoablated tubules and restored spermatogenesis. GFP tagged MSCs and Sertoli cells invariably always yielded non-green wild type sperm. Thus MSCs did not trans-differentiate, rather provided a healthy niche for VSELs to differentiate into sperm. Chemoablated mice could not mate and hence IVF was carried out using the sperm which resulted in early stage cleavage embryo.
Zhang et al., 2014 [50]	Allogeneic BMSCs were co-cultured in conditioned media derived from cultured testicular Sertoli cells *in vitro*, and then induced stem cells were transplanted into the seminiferous tubules of a busulfan-induced azoospermatic rat model for 8 weeks. Donor cells survived in recipient seminiferous tubules. Molecular markers of spermatogonial stem cells and spermatogonia (Vasa, Stella, SMAD1, Dazl, GCNF, HSP90α, integrinβ1, and c-kit) were expressed in the recipient testis tissue. No tumor mass, immune response, or inflammatory reaction developed. BMSCs might provide the potential to trans-differentiate into spermatogenic-like-cells, enhancing endogenous fertility recovery.
Cakici et al., 2013 [51]	Busulfan treated rats were injected GFP tagged MSCs via rete testes. After 12 weeks, spermatogenesis was detected in few tubules and GFP+/VASA+ and GFP+/SCP1+ cells in testes indicated the trans-differentiation of MSCs into spermatogenetic cells in the appropriate microenvironment. Rats withcell treatment were mated to show the full recovery of spermatogenesis, and continuous generations were obtained. GFP tagged sperm were noted in offspring also.
Monsefi et al., 2013 [52]	Donor MSCs from bone marrow were transplanted in busulphan (40 mg/Kg) treated rat testis. BrdU labeled MSCs (1.75×10^5^) were transplanted into the seminiferous tubules. The injected BrdU labeled MSCs differentiated to spermatogonia and spermatozoa in the seminiferous tubules of the infertile testis and also to the interstitial cells between tubules.
Sabbaghiet al, 2012 [53]	Rat bone marrow MSCs (5-10×10^6^ cells) were cultured and transplanted via rete testis into torsionedazoospermic testis. Germ cell specific markers (Oct4, Vasa and c-Kit) were monitored for the differentiation of MSCs after transplantation. They did not observe recovery of spermatogenesis.
Aziz et al., 2011 [54]	Bone marrow derived MSCs were transplanted into busulphan treated rats. One group received undifferentiated MSCs while another received transdifferentiated MSCs. Results showed that MSCs have potential for *in vitro* transdifferentiation into germ cells and in vivo transdifferentiation into spermatids and spermatocytes.
Lassalle et al., 2008 [55]	Total bone marrow cells were transplanted into the seminiferous tubules of germ cells deficient C57B16J mice after one month of busulphan treatment. Found no evidence for transdifferentiation of bone marrow cells into gametes.
Lue et al., 2007 [56]	Bone marrow cells from adult green fluorescent protein (GFP) mice were transplanted into seminiferous tubules and testicular interstitium of busulfan-treated wild-type or c-kit mutant (W/Wv) mice. 10–12 weeks after transplantation, cells were found to survive in recipient testes. Some GFP-positive donor cells appeared like Sertoli cell and expressed FSHR within the seminiferous tubules. GFP-positive cells in the interstitium expressed cytochrome P450 side chain cleavage enzyme (P450scc). Few GFP-positive donor cells had the appearance of spermatogonia or spermatocytes and expressed VASA. However, this was not found in the seminiferous tubules of W/Wv mice. Thus adult bone marrow cells, in a favorable testicular environment, differentiate into somatic and germ cell lineages. The resident neighboring cells in the recipient testis may control site-appropriate stem cell differentiation.

### Ovarian function restoration in cancer survivors

A total of 37 births have occurred till now by transplanting cryopreserved cortical tissue in cancer survivors [58]. Recently, Demeestere et al. [59] report spontaneous pregnancy and live-birth in a 27 years old survivor from cryopreserved ovarian cortical tissue which was cryopreserved as she suffered from sickle cell anemia at the age of 5 years. Four thawed ovarian fragments were grafted on the residual left ovary, six were grafted in the right peritoneal bursa, and five were grafted subcutaneously. Normal menstruation along with normal FSH and undetectable AMH levels was maintained after transplantation and the woman became pregnant spontaneously after more than 2 years of transplantation of cortical tissue. They reported that cortical transplantation led to successful recovery of ovarian function. Since other reports have shown spontaneous recovery of atretic ovary even without direct ovarian transplantation, *it is almost impossible to reason out source of oocytes that gave rise to the babies - whether it is cortical tissue or the ‘regenerated’ non-functional ovary.* Four years ago, Oktay and his team reported 4 spontaneous pregnancies and 3 live births after subcutaneous transplantation of cryopreserved ovarian tissue [42] in a 28 years old survivor of Hodgkin’s disease. Origin of the pregnancy was proposed to be due to improvement of function of *in situ* ‘menopausal’ ovary by hormones secreted by transplanted cortical tissue [60]. Oktay et al. [61] discussed a possible stem cell link to spontaneous pregnancy. Besides destroying follicles, oncotherapy also destroys the somatic niche and transplantation of cortical tissue provides ‘regenerative signals’ to intact non-functional ovary enabling oocyte formation from stem cells (source could be bone marrow) and hence pregnancy and live births.

Authors propose that it could be the surviving VSELs in non-functional ovary that become functional and undergo neo-oogenesis and primordial follicle assembly and these follicles then undergo normal maturation and ovulation resulting in spontaneous pregnancy and live births. These views are strongly supported by a recent study by our group on chemoablated mice ovaries wherein Sriraman et al. [8] found the VSELs survive chemotherapy, are located in the OSE and are functional since they self-renew in response to PMSG treatment (Table 1). Culture of intact chemoablated ovary in presence of FSH resulted in appearance of proliferating germ cell clusters *in situ* that incorporated BrdU and expressed MVH and STRA8. When enzymatically isolated OSE *in vitro* were cultured, germ cell nests that co-expressed PCNA and OCT-4 were observed. Prolonged culture showed differentiated GDF-9 and MVH expressing oocyte-like structures *in vitro*. The study underwent a very strict review and we were advised to publish 4 images of germ cell nests (in the Supplement) to convince the readers as it is very significant finding that postnatal ovary even after chemoablation retains stem cells which can form germ cell nests. These results are in contrast to those published by Lei and Spradling [62] who earlier failed to detect any evidence for presence of stem cells in adult ovary and we had argued that absence of evidence does not necessarily imply evidence for absence [63]. It is interesting to point out that (i) Lee et al. [64] showed that although bone marrow transplantation could rescue long-term fertility in chemoablated mice- but all the offspring were of recipient germline (ii) Niikura et al. [23] reported that stem cells in aged ovary can form follicles when exposed to a young niche of the host (iii) Edessy et al. [65] transplanted autologus mesenchymal cells isolated from bone marrow into the POF ovary resulted in resumption of menstruation and secretory changes in the endometrium and (iv) Zhu et al. [66] reported beneficial effect of transplanting human cord blood mesenchymal cells in rat chemoablated ovary. Table 3 shows various studies demonstrating beneficial effect of transplanting mesenchymal cells in chemoablated ovary associated with live births.

**Table 3:** Mesenchymal Cells to Restore Ovarian Function

Study	Highlights
Lai et al., 2015 [67]	Mouse ovaries were damaged by treatment with busulfan and cyclophosphamide. Human endometrial mesenchymal stem cells (EnSCs) were transplanted via tail vein. EnSCs transplantation increased body weight and improved estrous cyclicity as well as restored fertility. Reduced loss of germ stem cells pool. Live pups were born in the transplanted group.
Zhu et al., 2015 [68]	Human cord blood MSCs injected in cyclophosphamide treated rats resumed pregnancy. Direct injection into the ovary was better than tail vein injection. It led to resumption of estrus cycles, normal levels of sex hormones, restoration of fertility and normal offspring were born
Liu et al., 2014 [69]	Human Menstrual blood stem cells were injected into a cyclophosphamide-induced mouse model of POF. The transplanted cells survived in mouse ovaries for at least 14 days in vivo and the transplanted ovaries expressed higher levels of ovarian markers [AMH, inhibin α/β, and follicle-stimulating hormone receptor (FSHR)], and the proliferative marker Ki67]. Ovarian weight, plasma E2 level, and number of normal follicles increased over time in the HuMenSC group compared with the control group.
	Further, microarray analysis of cDNA expression patterns revealed that, after HuMenSC transplantation, the gene mRNA expression patterns in the ovarian cells following stimulation of the host ovarian niche became increasingly similar to those observed in human ovarian tissue compared with the pretransplantation mRNA expression pattern in HuMenSCs.
Terraciano et al., 2014 [70]	Female mice were treated with cisplatin to induce ovarian failure. Later GFP tagged ADSCs, FGSCs, or ovary cell suspension was transplanted in the ovary. This resulted in increased numbers of follicles and improved ovarian function.
Kilic et al., 2014 [71]	Female rats were treated with cyclophosphamide to induce ovarian failure. Bone marrow MSCs were transplanted along with cyclophosphamide to one group. Number of follicles were higher in the MSCs transplanted group.
Abd-Allah et al., 2013 [72]	Rabbits were treated with cyclophosphamide to induce ovarian damage. MSCs from male rabbit were transplanted through intravenous route. Increased follicle numbers with apparent normal structure of ovarian follicles were observed in the MSC recipient group
Guo et al. 2013 [73]	Bone marrow derived mesenchymal cells reduced rat granulosa cell apoptosis induced by cisplatin and age
*Ghadami et al., 2012* [74]	Bone marrow transplantation restored follicular maturation and steroid hormones production in a FORKO mouse model for primary ovarian failure. 24 h after transplantation, treated mice showed changes in vaginal smears and significant increase in both maturation and total number of follicles in treated animals. FSH dropped to 40–50 % and estrogen increased 4–5.5 times in the serum of treated animals compared to controls.
Fu et al., 2008 [75]	Bone marrow mesenchymal stem cells transplantation improved function of chemoablated rat ovaries. MSC released factors like VEGF, HGF and IGF-1 in vitro.

Just as in men, spontaneous pregnancy and birth of babies has been reported after BMT in women. Veitia et al. [76] observed spontaneous menstruation followed by pregnancy in a woman after 8 years of BMT, although she was expected to be infertile after suffering from Fanconi anemia and aggressive BMT conditioning regimen. Genetic analysis of the mother (patient), daughter and the donor revealed genetic relationship between the mother and daughter. As a result they ruled out that transplanted bone marrow was a source of oocytes. Similarly Nabhan et al. [77] also confirmed genetic relationship between the transplanted mother and daughter and excluded the possibility of germ cells transmission from the donor. We postulate that the transplanted bone marrow only helps to improve the niche (is not a source of germ cells) and endogenous VSELs differentiate into oocytes resulting in spontaneous birth of babies.

## Conclusions

To conclude, we have made a humble attempt based on available literature, to reason out that both ovary and testis rendered infertile by cancer treatment retain a novel stem cell population of VSELs that survive because of their quiescent nature and have the potential to regenerate the non-functional gonads when a healthy niche is provided. Authors opine that thus there may not be any need to remove ovary or testicular biopsy in children/adults prior to cancer therapy for later transplantation and this will result in major paradigm shift in the field of oncofertility. The non-functional ovaries and testis in young cancer survivors could be regenerated by transplanting mesenchymal niche cells which will secrete trophic factors required for the surviving VSELs to differentiate into gametes. This will help them in normal development of secondary sexual characteristics, avoid hormone replacement therapy, normal menstrual cycles and they may plan parenthood whenever they desire. Our reasoning in this article has huge translational potential and will greatly help improve quality life of cancer survivors. We are open to anyone who is interested to visit our lab to observe and be convinced by the presence of VSELs in chemoablated testis and ovary.

## End notes

1. Adult mammalian ovary and testis harbor two populations of stem cells including pluripotent VSELs which are relatively quiescent and undergo asymmetric divisions to self-renew and give rise to the progenitors which comprise spermatogonial stem cells (SSCs) in testis and ovarian stem cells (OSCs or OGSCs) in ovaries. This stem cells activity in the ovary surface epithelium and testicular basal seminiferous tubular epithelium maintains life-long homeostasis in the gonads.

2. Being quiescent, VSELs survive oncotherapy as the cancer treatment targets actively dividing cells. Several reports suggest that even the niche gets compromised by oncotherapy and thus cannot support (provide growth factors and cytokines) VSELs to differentiate and form gametes. Studies in mice and circumstantial evidence in humans suggest that when chemoablated testis and ovary are exposed to a healthy microenvironment, VSELs differentiate into healthy gametes and result in live pregnancies.

3. Differentiation of ES or iPS cells *in vitro* into gametes is not yet successful as they do not easily convert into PGCs whereas the VSELs (considered equivalent to PGCs) isolated from normal and chemoablated gonads spontaneously differentiate into sperm and oocyte-like structures [7, 8, 78]. The underlying reasons for better potential of VSELs compared to ES/iPS cells to produce ‘synthetic gametes’ were recently discussed [79].

4. There may be no need to cryopreserve testicular and ovarian tissue prior to cancer treatment. Urgent pilot clinical trials (transplantation of mesenchymal cells directly into chemoablated gonads) need to be undertaken to provide a strong basis to our viewpoint. Meantime, if testicular tissue is collected for cryopreservation, it may be ideal to expand Sertoli cells in culture and cryopreserve for future use in addition to the germ cells.

## Abbreviations

BMT: Bone marrow transplantation
ES cells: Embryonic stem cells
iPS cells: Induced pluripotent stem cells
MSCs: Mesenchymal stem cells
OGSCs: Ovarian germ stem cells
OSCs: Oogonial stem cells
OSE: Ovary surface epithelium
PGCs: Primordial germ cells
POF: Premature ovarian failure
SSCs: Spermatogonial stem cells
VSELs: Very small embryonic-like stem cells
